# Hematopoietic stem cell transplantation in a newborn suffering from severe combined immunodeficiency and severe hemophilia A: a case report and review of the literature

**DOI:** 10.1016/j.rpth.2025.102842

**Published:** 2025-03-30

**Authors:** Sarah Schober, Michaela Döring, Peter Lang, Johannes Schulte, Martin Olivieri, Vanya Icheva

**Affiliations:** 1 Department I–Haematology, Oncology, Gastroenterology, Nephrology, Rheumatology, University Children’s Hospital Tuebingen, Tuebingen, Germany; 2 Paediatric Thrombosis and Haemostasis Unit, Paediatric Haemophilia Center, Dr. von Hauner Children’s Hospital, Ludwig Maximilian University Clinic, Munich, Germany; 3 Department II–Paediatric Cardiology, Intensive Care Medicine and Pulmonology, University Children’s Hospital Tuebingen, Tuebingen, Germany

**Keywords:** case reports, hematopoietic stem cell transplantation, newborn, severe combined immunodeficiency, hemophilia A

## Abstract

**Background:**

Severe combined immunodeficiency (SCID) and severe hemophilia A are 2 rare and potentially life-threatening congenital diseases. The coincidence of these diseases has not been reported so far.

**Key Clinical Question:**

We present the first case of a newborn with both diseases. SCID can be treated with hematopoietic stem cell transplantation (HSCT). However, how to successfully manage a newborn with severe hemophilia A during intensive HSCT treatment is the key clinical question of this case report.

**Clinical Approach:**

Prophylactic factor (F)VIII substitution during HSCT was performed with an extended half-life FVIII product (efmoroctocog alfa). The platelet count was a major factor influencing the dosage of FVIII. No bleeding complications or FVIII inhibitors occurred during this individualized management.

**Conclusion:**

This is the first case report of a newborn suffering from both SCID and severe hemophilia A. HSCT is feasible in this situation without bleeding complications if an individual substitution regimen with FVIII is applied.

## Essentials

• Severe hemophilia A and severe combined immunodeficiency are rare, life-threatening diseases.
• For the first time, a newborn with both diseases requiring stem cell transplantation is described.
• Stem cell transplantation is feasible with individual coagulation management.
• An overview of all published cases of HSCT in persons with hemophilia is provided.

## 1 Introduction

Today, hemophilia can be treated, resulting in normal life expectancy and low bleeding risk in daily life [1,2]. However, life-threatening bleeding can occur in the event of severe trauma or in combination with other diseases. Furthermore, development of factor (F)VIII inhibitors is a very serious complication [[1], [2], [3]]. The risk of developing an inhibitor depends on various causes and is highest during the first 50 days of factor exposure [3]. Aside from the underlying genetic mutation, the risk is also increased if higher dosing is required during the first exposure days, as in severe trauma or surgery. Finally, inflammation caused, for example, by infections or surgery and the associated immune reactions might also play a role in inhibitor development (“danger theory”) [4].

Severe combined immunodeficiency (SCID) is a rare congenital disease with an estimated incidence of 1:62,500 infants in Germany [5]. SCID combines a heterogeneous group of life-threatening monogenetic disorders with an impaired immune system and variable clinical features. SCID-affected infants present with severe infections, failure to thrive, and possibly skin anomalies or diarrhea. Without suitable treatment, these children die within the first year of life. Thus, early diagnosis and treatment are essential [6]. Today, mortality in SCID is still high and stated at around 29% [5]. Postnatal isolation, strict hygiene, and supportive anti-infective prophylaxis are crucial in preparation for therapy. Both hematopoietic stem cell transplantation (HSCT) and gene therapy are potentially curative therapies [7,8]. Since gene therapy is still experimental, HSCT is the recommended therapy up to now [9].

A side effect of induction chemotherapy is pancytopenia, resulting in high risks for infection, anemia, and bleeding.

So far, no person suffering from both severe hemophilia and SCID has been reported. Information on HSCT in persons with hemophilia is scarce. For the first time, this case report describes the unique situation of a newborn with these 2 orphan congenital diseases and answers the key clinical question: how to successfully manage such a patient during HSCT despite the inherent and acquired bleeding risks. Furthermore, this report provides a review of all published cases of HSCT in hemophilia.

## 2 Case Report

A male newborn was admitted due to a pathological newborn screening test suspecting SCID at the age of 15 days.

The boy presented in good condition without any complaints or infections. He was born at term and showed good drinking habits and adequate levels of activity. The physical examination and body measurements were age-appropriate without any pathologies.

The healthy parents are consanguineous (cousins) and originate from Syria. They have 3 more children, 2 healthy girls and 1 boy suffering from severe hemophilia A. The older brother was 16 years old when the boy was born. He experienced several severe joint bleeds and did not receive any factor prophylaxis until he was 9 when he moved from Syria to Germany. As a result, he suffers from chronic joint damage. There are no inhibitors present in the older brother. There are no known SCID cases in the extended family.

The boy was admitted to the HSCT unit and isolated immediately. The following diagnostics showed normal blood count: white blood cells: 2600/μL; 53.8% neutrophils; 33.1% lymphocytes; 13.1% monocytes; hemoglobin: 9.8 g/dL; platelets: 405,000/μL; and immunoglobulins (Igs) IgG: 644 mg/dL and IgA < 6 mg/dL. Flow cytometry revealed T and B cell lymphocytopenia with CD3 80/μL, CD19 1/μL, CD4 52/μL, CD8 8/μL, and CD16/56 1074/μL. In the chimerism analysis, no maternal T or B cells were detected. T-cell receptor repertoire analysis showed a T-cell developmental disorder with an oligoclonally altered V beta repertoire. Thus, with CD3 T cells less than 300/μL and low T-cell function, a typical SCID T-/B-/NK+ Stratum A was suspected [10]. Genetic analysis revealed the underlying pathogenic homozygous *RAG1* missense variant (c.2095C>T, p. Arg699Trp, and OMIM∗179615) causing SCID and confirmed the need for HSCT.

Based on the brother’s severe hemophilia A, activated partial thromboplastin time and FVIII levels were tested in the newborn (activated partial thromboplastin time: 82 seconds, FVIII < 0.5%). Severe hemophilia A was confirmed via genetic testing, which revealed a hemizygous intron 22 inversion (NG_011403.1, NM_000132.3). Clinically, there were no signs of bleeding so far. However, the combination of SCID and severe hemophilia A complicated the HSCT process.

To start HSCT, a central line was required. At the age of 3 months, a Hickman catheter was surgically implanted (d-9 pre-HSCT). Perioperative factor replacement therapy was performed using an extended half-life (EHL) FVIII product (efmoroctocog alfa). The patient received 88 IU/kg before and 44 IU/kg after the intervention and on days 1, 3, 5, and 7. Additionally, a tranexamic acid continuous infusion of 2 mg/kg/h was applied perioperatively until day 4. No bleeding occurred with this substitution regime. HSCT was performed after myeloablative conditioning at the age of 3 months because no matched-related donor was available. The boy received T-cell-depleted peripheral stem cells from a matched (9/10) unrelated donor (30 × 10^6^/kg bodyweight CD34+ cells). During the initial phase of HSCT, FVIII was supplemented twice weekly with 40 IU/kg. The conditioning phase and the subsequent aplasia were the most critical periods concerning the bleeding risk due to thrombocytopenia. The platelet transfusion threshold was set at 40,000/μL. In total, the patient received 4 platelet transfusions. The coagulation management was adapted to the platelet count. During the aplastic period (d+6 to d+16) with a platelet count < 100,000/μL, factor substitution was individually adjusted to achieve FVIII trough levels >50% (40 IU/kg daily to every other day). After hematopoietic reconstitution with a platelet count > 100,000/μL, FVIII substitution was reduced to 38 IU/kg twice a week, and finally, in the absence of bleeding symptoms, to once a week in accordance with the standard protocol of our center for this age group. Due to prolonged inpatient treatment, the patient was less physically fit compared with other children of the same age and had developed muscle hypotonia; thus, there was no need for increased factor substitution. With this individualized management (Figure), there were no bleeding complications, and no inhibitor was detected. There were no relevant side effects during HSCT, and the following immune reconstitution took place accordingly. Two years after HSCT, no inhibitor and no relevant bleeding has been reported in the patient receiving the same EHL product prophylaxis (2 × 68 IU/kg/wk).

**Figure:**
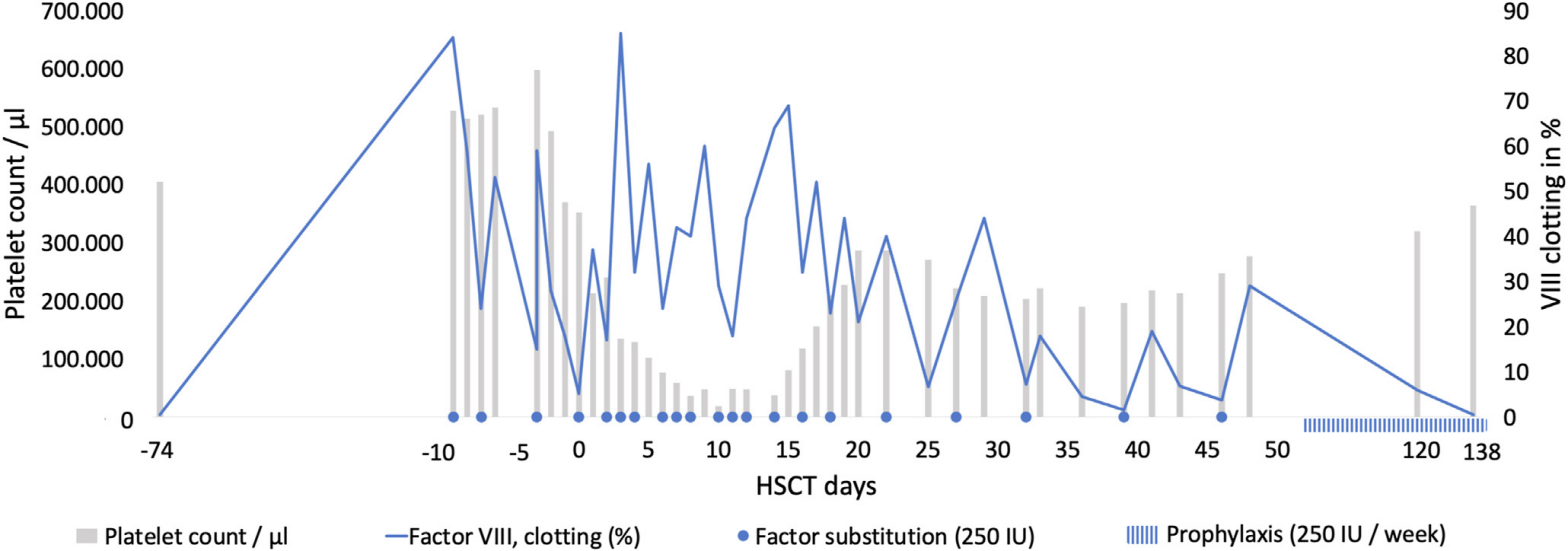
Coagulation management during hematopoietic stem cell transplantation (HSCT). IU, International Units.

## 3 Discussion and Review of the Literature

This is the first case of a newborn suffering from SCID and severe hemophilia A. This report demonstrates that HSCT is feasible in this situation without bleeding complications if a personalized substitution regimen is applied based on the platelet count. In general, little is known about HSCT in persons with hemophilia, especially in newborns.

There are 11 cases (including this case) reporting on HSCT in persons with hemophilia in the literature [[11], [12], [13], [14], [15], [16], [17], [18], [19], [20]]. Seven of these reports are on pediatric patients. In 8 patients, severe hemophilia was present. In 7 of the 11 patients, a malignant underlying disease was the cause of HSCT. In 4 patients, autologous HSCT was performed. The others received allogeneic transplantations. In 2 patients, a sibling donor was present (Table).

**Table:** Review of all published cases of hematopoietic stem cell transplantation in persons with hemophilia.

Patient	Condition	Type of hemophilia	Transplantation	Coagulation management during HSCT	Outcome	Ref.
Male, 3 mo	SCID	Severe A	Allogeneic, PBSCs, matched-unrelated donor	If platelets <100,000/μL, EHL rFVIII 40 IU/kg daily to every other day to achieve FVIII levels >50%, tranexamic acid during operation	Alive and well, 2 y after HSCT	This case
Male, 8 mo	Osteopetrosis	Mild (16%) A	Allogeneic, cord blood, unrelated donor	pFVIII 50-75 IU/kg 1×/d, 2×/wk after HSCT. VOD: switch to rFVIII, threshold platelet transfusion < 25,000/μL	No hemorrhages, impairment due to osteopetrosis, several interventions: ventriculostomy, ventriculoperitoneal shunt, liver biopsy, tracheostomy	[11]
Male, 11 mo	Neuroblastoma	Severe B	Autologous	Pre-HSCT paraneoplastic coagulopathy, tumor bleeding: FFP, FXIII, pFIX (up to 50 IU/kg 3×/d), tranexamic acid, vitamin K. During HSCT: switch from pFIX to EHL rFIX 50 IU/kg 3×/wk. FIX trough level >30%, peak <150%	Pre-HSCT: paraneoplastic coagulopathy, tumor hemorrhage, no hemorrhage during HSCT, CNS relapse with cerebral hemorrhages 15 mo after initial diagnosis	[12]
Male, 19 mo	Neuroblastoma	Severe A	2 × autologous (tandem)	rFVIII 25 IU/kg 3×/wk up to rFVIII 40 IU/kg q8h depending on hemorrhagic risk. Threshold platelet transfusion < 50,000/μL	No hemorrhages	[13]
Male, 4 y	MDS-RAEB	Mild (19%) A	Allogeneic, bone marrow, matched-unrelated donor	Procedures: FVIII 50 IU/kg/d before, followed by 4 × 30 IU/kg/d for 1 d, 3 × 30 IU/kg/d for 4 d, during HSCT FVIII levels 25%-50%; after HSCT 2 × 30 IU/kg/d. Threshold platelet transfusion < 30,000/μL	No hemorrhages, alive and well, 1.5 y post-HSCT	[14]
Male, 7 y	Aplastic anemia	Moderate (3%)	Allogeneic, bone marrow, matched sibling donor	Procedures prior to HSCT: platelet transfusion < 50,000/μL, rFVIII 60 IU/kg, 2 × 30 IU/kg for 3 d. During HSCT: threshold platelet transfusion < 30,000/μL rFVIII substitution on demand	Hemorrhages at catheter site, alive and well, 4 mo after HSCT	[15]
Male, 14 y	ALCL	Severe A	Allogeneic, cord blood, matched-unrelated donor	Threshold platelet transfusion < 30,000/μL, 2× rFVIII 1000 IU/d	No hemorrhages, alive and well 3 y after HSCT	[16]
Male, 26 y	HIV, sec. Burkitt type ALL	Severe	Allogeneic, bone marrow (HIV-positive)	N/A	Died after CNS relapse, 18 mo after HSCT	[17]
Male, 38 y	Mantel cell lymphoma, chronic hepatitis B, C	Severe A	Autologous	Prior HSCT: platelets < 50,000/μL, pFVIII 40 IU/kg daily, levels FVIII 17% to 35% (after 12 h). During HSCT: 2 × 20 IU/kg/d, pFVIII >40%; platelets < 30,000/μL: 2 × 30 IU/kg/d, pFVIII >50%, threshold: platelet transfusion < 20,000/μL	No hemorrhages	[20]
Male, 36 y	AIDS-related plasmablastic lymphoma	Severe A	Autologous	During therapy: outpatient rFVIII 20 IU/kg/d, inpatient continuous infusion rFVIII, levels > 40% (100% in sepsis, procedures), threshold: platelet transfusion < 20,000/μL	No hemorrhages, relapse, died 14 mo after initial diagnosis	[19]
Male, 22 y	FVIII inhibitor	Severe A	Allogeneic, bone marrow, matched sibling donor	pFVIII, rVIIa, FEIBA, tranexamic acid	Soft tissue hemorrhages, died of sepsis d+46, inhibitor present	[18]

ALCL, anaplastic large cell lymphoma; ALL, acute lymphoblastic leukemia; CNS, central nervous system; EHL, extended half-life; FEIBA, anti-inhibitor coagulant complex; FFP, fresh frozen plasma; FIX/FVIII/FXIII, factor IX/VIII/XIII; HSCT, hematopoietic stem cell transplantation; IU, International Units; MDS, myelodysplastic syndrome; N/A, not available; PBSC, peripheral blood stem cell; pFVIII, plasma-derived factor VIII product; pFIX, plasma-derived faktor IX product; q8h, every 8 hours; RAEB, refractory anemia with excess blasts; Ref., reference; rFVIII, recombinant factor VIII product; rFIX, recombinant factor IX product; rVIIa, recombinant activated factor VII; SCID, severe combined immunodeficiency; sec., secondary; VOD, veno-occlusive disease.

In summary, there were no severe hemorrhages described in the context of HSCT in persons with hemophilia. All patients received a personalized prophylactic factor replacement regimen adjusted to the individual bleeding risk. So far, there is no standard replacement protocol. Thus, every patient was treated differently (Table). In some patients, factor dosage depended on the platelet count or trough factor levels; in others, on procedural or bleeding risks. Some patients had elevated platelet transfusion thresholds (20,000-50,000/μL). One patient was treated with continuous factor infusion [19].

The presented patient’s treating physicians considered the option of emicizumab but ultimately decided to only treat the patient with an EHL factor product due to concerns about potential drug interactions and the unclear risk of microangiopathy in the context of HSCT.

In the presented case, as well as in 2 other reports, tranexamic acid was applied in addition to factor replacement therapy [12,18].

None of the patients described in the literature developed any inhibitor following HSCT. One patient was transplanted in an attempt to treat an inhibitor but died of side effects [18].

So far, the patient presented here has not developed any FVIII inhibitor despite high doses of substituted FVIII. Intron 22 inversion is associated with a high inhibitor risk of about 35% [1]. It could be assumed that the risk of developing inhibitors is lower in a patient with SCID due to the impaired immune system. After allogeneic HSCT, the inhibitor risk presumably remains lower since the healthy donor cells were exposed to normal FVIII levels. However, this is speculative, and time will tell how the case evolves with a maturing immune system.

Today, HSCT is the standard of care for SCID patients. Nevertheless, this treatment is associated with several risks and side effects. Gene therapy might be the treatment option of the future. Unlike HSCT, the advantage of gene therapy in SCID is to avoid high-dose chemotherapy, along with its associated toxicity and side effects [6].

Waiving the need for HSCT would be an excellent option considering the significant HSCT-associated bleeding risk due to liver toxicity, veno-occlusive disease, hemorrhagic cystitis, or delayed engraftment. Around 25% of HSCT patients suffer moderate to severe hemorrhages [11].

In future, gene therapy may become a potential cure for SCID and a viable treatment option for pediatric severe hemophilia. However, it is unlikely to be suitable for infants or young children suffering from hemophilia, and may be more realistic for teenagers.

The data presented in this case report do not allow any generalization concerning treatment recommendations or cause-effect relationships as this is a retrospective description of one patient suffering from 2 extremely rare monogenetic diseases but can serve as a template for similar cases since this coincidence has not been described before. Managing hemophilic patients during HSCT remains a challenge and has only been described in a few individual case reports. As life expectancy of persons with hemophilia has normalized, coincidence of hemophilia and malignancies with the need for HSCT will be more frequent. Thus, a standardized coagulation management regimen for persons with hemophilia undergoing HSCT would be beneficial for best treatment and outcome.
